# *Haematococcus lacustris* Carotenogensis: A Historical Event of Primary to Secondary Adaptations to Earth’s Oxygenation

**DOI:** 10.3390/life14050576

**Published:** 2024-04-30

**Authors:** Cui Lan Qu, Hui Jin, Bing Zhang, Wei Jian Chen, Yang Zhang, Yuan Yuan Xu, Rui Wang, Yong Min Lao

**Affiliations:** 1 Shenzhen Institute of Quality & Safety Inspection and Research, Shenzhen 518055, China; 2 Southern Marine Science and Engineering Guangdong Laboratory, Guangzhou 511458, China; jin_hui@gmlab.ac.cn; 3 Institute for Advanced Study, Shenzhen University, Shenzhen 518060, China

**Keywords:** *Haematococcus lacustris*, lycopene cyclase, oxygenation, green lineage, carotenoid biosynthesis, adaptation

## Abstract

(1) Background: Oxygen has exerted a great effect in shaping the environment and driving biological diversity in Earth’s history. Green lineage has evolved primary and secondary carotenoid biosynthetic systems to adapt to Earth’s oxygenation, e.g., *Haematococcus lacustris*, which accumulates the highest amount of secondary astaxanthin under stresses. The two systems are controlled by lycopene ε-cyclase (LCYE) and β-cyclase (LCYB), which leave an important trace in Earth’s oxygenation. (2) Objectives: This work intends to disclose the underlying molecular evolutionary mechanism of Earth’s oxygenation in shaping green algal carotenogensis with a special focus on lycopene cyclases. (3) Methods: The two kinds of cyclases were analyzed by site-directed mutagenesis, phylogeny, divergence time and functional divergence. (4) Results: Green lineage LCYEs appeared at ~1.5 Ga after the first significant appearance and accumulation of atmospheric oxygen, the so-called Great Oxygenation Event (GOE), from which LCYBs diverged by gene duplication. Bacterial β-bicyclases evolved from β-monocyclase. Enhanced catalytic activity accompanied evolutionary transformation from ε-/β-monocyclase to β-bicyclase. Strong positive selection occurred in green lineage LCYEs after the GOE and in algal LCYBs during the second oxidation, the Neoproterozoic Oxygenation Event (NOE). Positively selected sites in the catalytic cavities of the enzymes controlled the mono-/bicyclase activity, respectively. Carotenoid profiling revealed that oxidative adaptation has been wildly preserved in evolution. (5) Conclusions: the functionalization of the two enzymes is a result of primary to secondary adaptations to Earth’s oxygenation.

## 1. Introduction

**Figure 1:**
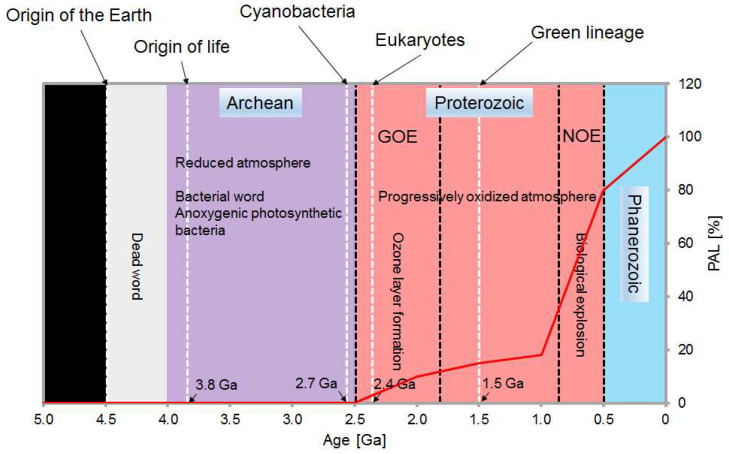
Timecourse of Earth’s oxygenation and accompanied biogenesis. The timecourse of Earth’s oxygenation and life is inferred according to our analyses and reported data [2,3,4,5,6,7]. Purple: Archean; red: Proterozoic; light blue: Phanerozoic; red line: present atmospheric level of oxygen (PAL, %); GOE: the Great Oxygenation Event; NOE: the Neoproterozoic Oxygenation Event; Ga: gigaannum.

During the history of the Earth (Figure 1), oxygen has had a great effect in shaping the planet’s environment and in driving biological diversity [1]. Likely, the first organisms on Earth at least 3.8 Ga [2] ago emerged and lived at very high temperatures under a reducing atmosphere without oxygen [3]. However, the appearance of oxygenically photosynthesizing cyanobacteria from 3.0 to 2.7 Ga triggered the first significant appearance and accumulation of atmospheric oxygen, the so-called Great Oxygenation Event (GOE) between 2.4 and 2.0 Ga [4,5]. During the GOE, the atmospheric oxygen concentration elevated from negligible levels to ~10% of the present atmospheric level (PAL) [6]. After, a relatively stable period (Mesoproterozoic) lasted from 1.85 to 0.9 Ga with poorly constrained oxygen levels ranging from 5% to 18% [4]. During this period, the first oxygenic photosynthetic eukaryotes, an ancient green lineage of green algae and plants, occurred at ~1.5 Ga by primary endosymbiosis, in which a heterotrophic eukaryote swallowed a cyanobacterium and turned it into a plastid [7]. Soon after the emergence of green lineage, a second oxidation, referred to as the Neoproterozoic Oxygenation Event (NOE), appeared at 0.9 to 0.5 Ga, accompanied by a steep increase in oxygen levels to near 80% PAL [4].

Elevated oxygen levels on Earth’s atmosphere have two opposite effects on life. The development of an ozone layer acts as a protective screen against harmful light radiation, which is presumed to have formed during the GOE [4]. On the other hand, intensive oxygenation generates reactive oxygen species (ROS) that potentially injure DNA, protein, lipid, and many biological functions. The problem could be worse because Earth’s atmosphere at that time had not yet fully developed, leading to a strong radiative environment [8]. Even after the ozone layer had formed, short-term changes in ozone layer abundance caused by active supernovae and volcanism resulted in a much higher light radiation than that of today [9]. The situation for green lineage could be more severe for its primary antioxidant systems, mainly referring to the plasmids obtained by endosymbiosis from cyanobacteria, had not yet adapted to or coped with the steep increase in oxygen contents at the beginning of the NOE. Extremely oxidative pressure urged green lineage to develop more efficient antioxidant systems, such as an enhanced secondary carotenoid biosynthetic pathway, based on the primary one.

According to the biological functions, carotenoids are classified into primary and secondary carotenoids. Primary carotenoids, including lutein and lycopene, are structural and functional components of the photosynthetic apparatus [10]. They are located within the thylakoid membrane to assist light harvesting, to quench free radical chlorophylls, and to dissipate excess light energy [10]. Secondary carotenoids such as canthaxanthin and astaxanthin are synthesized in a large amount via carotenogenesis only when cells are exposed to environmental stresses [11]. Astaxanthin is the most powerful antioxidant, 10 times greater than β-carotene, lutein, zeaxanthin, and canthaxanthin [12]. Secondary carotenoids are accumulated in lipid bodies out of the thylakoid membrane to protect cells from oxidative damage when the amount of primary carotenoids is not enough [13].

The biosynthetic pathway of carotenoids divides into two parts according to whether the products contain oxygen. Steps toward lycopene constitute the most common part among all photosynthetic organisms, which synthesize oxygen-free carotenes. Acyclic lycopene is then differentially cyclized to synthesize branch products. The α-branch point to primary carotenoids biosynthesis is controlled by lycopene ε-cyclase (LCYE), while the β-branch point to secondary carotenoids biosynthesis is governed by lycopene β-cyclase (LCYB). The cyclization of lycopene by lycopene cyclases marks an important regulatory point in carotenoid biosynthesis. The ε-cyclization controlled by LCYE is a key point in regulating primary carotenoid levels and the ratio of primary to secondary carotenoids [14].

*Haematococcus lacustris* surpasses any other reported sources to massively accumulate astaxanthin, up to 8% dry cell weight (DCW) [15], under environmental stresses [16]. The success of *H. lacustris* is attributable to their ability to maintain photosynthetic activity under increasing oxidative stress in the history of Earth’s oxygenation. In particular, *H. lacustris* evolved an enhanced secondary carotenoid biosynthetic pathway as sunscreen to protect the light-harvesting complexes (LHC) composed of primary carotenoids bound with chlorophylls [10], by dissipating excess light energy and shielding the photosynthetic apparatus, and as an antioxidant and physicochemical barrier against photodynamic damage by UV radiation [17]. We propose that increasing oxygen levels on the primitive Earth exerted a strong selective pressure on green lineage whose primary carotenoid biosynthetic system has been inherited from cyanobacteria by endosymbiosis, to attain an enhanced secondary carotenoid biosynthetic system, giving rise to the excellent ability of *H. lacustris* to massively accumulate astaxanthin against oxidative stress.

## 2. Materials and Methods

### 2.1. Bacterial Strain and Cultivation Conditions

The *E. coli* strains were cultured in LB medium containing 100 μg mL^−1^ ampicillin at 30 °C in dark with shaking at a speed of 220 rpm for 48 h. Then, the cells were transferred to a back chamber to completely induce carotenoid synthesis at 25 °C for another 48 h.

### 2.2. Plasmid Construction and Site-Directed Mutagenesis

The plasmid pACCRT-EIB was used for site-directed mutagenesis, which harbors *crtE*, *crtI*, and *crtB* from *Pantoea ananatis* encoding GGPP synthase (GGPPS), phytoene desaturase (PDS), and phytoene synthase (PSY), respectively, and confers the accumulation of lycopene to *E. coli* strains [18]. The open reading frames (ORFs) of *HlLcyB* and *HlLcyE* genes were isolated from *H. lacustris* by RT-PCR in our previous work [19] and, in this study, inserted into the *Hind*III site of the plasmid pACCRT-EIB to produce plasmids pACCRT-EIB-B and pACCRT-EIB-E, respectively. The codons of HlLCYE were optimized to those of *E. coli* for all functional assays here. Key amino acid residues that were detected by positive selection analysis in the catalytic caves of *HlLcyE* and *HlLcyB* were mutated, including L_457_, A_468_, V_531_, and G_370_ in HlLCYE, and H_376_, P_337_, and GTAX_3_HP in the cyclase motif 2 of HlLCYB, using the Transformer^TM^ Site-Directed Mutagenesis Kit (Clontech, Mountain View, CA, USA) according to the manufacturer’s instructions. All insertions were carried out using the In-Fusion^®^ HD Cloning Kit (Clontech, Mountain View, CA, USA) according to the user’s manual. All cloning primers were designed following the instructions of the kit. Primers used in this article are listed in Supplementary Table S1.

### 2.3. Pigments’ Extraction and UPLC Analysis

Pigments’ extraction, saponification and UPLC analysis were carried out according to the work of Jin et al. [20]. In brief, ~150 mL of *E. coli* DH5α cells was harvested by centrifugation at 12,000× *g* for 5 min at 4 °C, and incubated in a water bath at 55 °C for 15 min with vigorous shaking at a 5 min interval after the addition of 3 mL of acetone. Then, the supernatants were collected by centrifugation at 12,000× *g* for 15 min at 4 °C, and were subsequently evaporated to dryness and dissolved in 2 mL of acetonitrile. Then, 100 μL of extracts was diluted with 2 mL of acetonitrile and hydrolyzed by adding 10 μL of 1 M NaOH. Saponification was carried out for 6 h at 4 °C in the dark. The saponified extracts were then washed several times with distilled water until the pH was neutral, and were analyzed by UPLC directly.

A Waters ACQUITY UPLC™ H-CLASS equipped with a quaternary pump, an autosampler, a column oven and a PDA detector was used for carotenoid profiling. Carotenoids were separated at a flow rate of 0.4 mL min^−1^ on a Waters BEH C18 column (2.1 × 50 mm, 1.7 μm) using methanol (A) and acetonitrile (B). The gradient elution was 10% A, 90% B at 0 min, followed by a linear gradient to 0% A and 100% B to 4 min, maintained at 0% A and 100% B to 12 min, returned to the initial condition by 12.1 min, re-equilibrated at the initial condition by 15 min. The injection volume was 5 μL. The needle was washed using a acetonitrile/methonal (9:1; *v*/*v*) mixture for 10 s after each injection. Column temperature was maintained at 35 °C using a column oven. The detection of analysts was carried out by ultraviolet (UV) absorbance at 450 nm. The filter constant was set to 0.2. All system controls and data analyses were processed by the Empower 3.0 software.

### 2.4. Phylogenetic Tree Construction

Multiple alignments were conducted using Clustal X 2.1. A phylogenetic tree of 25 amino acid sequences was constructed by Bayesian interference with MrBayes 3.2.6 under the mixed substitutions. Two parallel runs were performed for 10 million generations, each run with four chains, including three heated chains and one cooled chain. Trees were sampled every 100 generations and with a burn-in of 2500 generations. The Neighbor Joining tree was also constructed by the MEGA 7.0 software [21]. Bootstrap values were estimated (with 1000 replicates) to assess the relative support for each branch, and bootstrap values were labeled with cutoff = 50.

### 2.5. Divergence Time Analysis

Based on the Bayesian tree, a time tree was constructed by the MEGA7 software using the Reltime method and the General Time Reversible model [22]. The time tree was computed using DrLCYBm-AmCrtY node as calibration with constraints ranging from 2.696 to 3.035 billion years, and AaLCYE-CzLCYB node from 1.244 to 1.6 billion years. A discrete Gamma distribution was used to model evolutionary rate differences among sites (5 categories (+G, parameter = 0.7823)). All positions containing gaps and missing data were eliminated. The bacterial cyclases were used as the outgroup.

### 2.6. Recombination Detection

For recombination detection, the RDP 4.0 software was run using default parameters [23]. Only those that are significantly detected by at least four detection methods are considered candidates of recombination.

### 2.7. PAML Analysis

Finally, selection analysis using the subtree was performed by the Codeml program implemented in the PAML 4.9 software [24]. Paired comparison of site-specific models, such as discrete model M3 and one-ratio null model M0, selection model M2a and neutral null model M1a, beta and ω model M8 and beta null model M7, was carried out. Then, pairs of branch-specific models including a free-ratio model (M1) and a one-ratio model (M0), two-ratio models (Ta-Ti) and one-ratio model (M0) were compared (Ta/M0, Tb/M0, Tc/M0, Td/M0, Te/M0, Tf/M0, Tg/M0, Th/M0, and Ti/M0). Finally, pairs of branch-site models (Ab/A1b, Af/A1f, Ag/A1g, Ah/A1h, and Ai/A1i) were compared to further test positive selection on amino acid sites in specific branches. Sequence data from this article can be found in Supplementary Table S2.

### 2.8. Functional Divergence Analysis

Functional divergence was performed using DIVERGE 3.0 [25].

### 2.9. Sites Mapping

The 3D structures of HlLCYE and HlLCYB were constructed by the homology-modeling server SWISS-MODEL (https://swissmodel.expasy.org/ (accessed on 27 March 2023)) [26] using *Sulfolobus acidocaldarius* geranylgeranyl reductase (GGR) (4opl.1.A) as the template. Sites under positive selection were then mapped onto the structures by Chimera 1.11 [27].

### 2.10. Statistical Analysis

The data were processed by one-way analysis of variance using SPSS version 13.0. Summary statistics were expressed as means ± standard deviations (SD). In all statistical analyses, *p* < 0.05 was considered statistically significant.

## 3. Results

### 3.1. Phylogenetic Analysis of Cyclases

**Figure 2:**
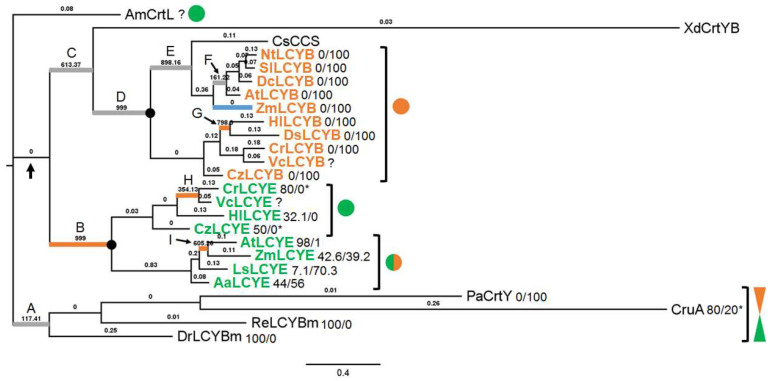
Bayesian phylogenetic tree of cyclases. Thick arrow shows the primary endosymbiosis event. Bold filled dots indicate green lineage LCYEs and LCYBs. Color-filled cycles indicate the ratio of activity between mono- and bicyclase: green cycle for monocyclase, orange cycle for bicyclase, and mixed color cycle for mixed activities. Mixed color funnel indicates a trend of activity transformation in bacterial cyclases, green part represents monocyclase activity, and yellow part represents bicyclase activity. Enzyme activity is given as molar ratio of mono-/bicyclase, according to reports and our analysis (detail in text). Asterisk indicates the peak area ratio of mono-/bicyclase of the enzyme roughly estimated by us, according to the assay reported. Question mark indicates that the activity of the enzyme is unknown. Branches under positive selection (ω > 1) showing statistically significant results are highlighted by thick orange lines, while those do not show statistically significant results are highlighted by thick grey lines; branches under recombination predicted by RDP4 (Supplementary Table S4) are shown as thick blue line. The estimated ω ratios are given above the branches.

Previously, we had characterized a missing HlLCYE and compared the cyclase activities of HlLCYB and HlLCYE in *H. lacustris* [19]. We found that HILCYE is a ε-monocyclase capable of synthesizing ε-monocyclic carotenoids like α-zeacarotene and δ-carotene, while HlLCYB can synthesize β-bicyclic carotenoids such as β-carotene [19]. Based on these findings, the phylogenetic relationship of ε-monocyclase and β-bicyclase was investigated here. Phylogenetic reconstruction by the Neighbor Joining method (Supplementary Figure S2) and Bayesian inference (Figure 2) gave similar topologies, but the Bayesian algorithm has generated higher support values at all branches and thus was used. As Figure 2 illustrates, a clearly evolutionary order was reconstructed by Bayesian inference using cyclases from anoxygenic photosynthetic bacteria, cyanobacterium, fungus, algae, and plants. In this tree, bacterial β-cyclases first evolved; then, cyanobacterial β-cyclase emerged, which subsequently diversified into eukaryotic cyclases. Though all green lineage cyclases evolved from the plant-type cyanobacterial CrtL, LCYEs seemed to evolve first; then, their LCYB paralogs arose probably by gene duplication (Figure 2). Notably, bacterial bicyclases (e.g., PaCrtY and CruA) seemed to evolve from monocyclases (e.g., DrLCYBm and ReLCYBm), while plant LCYEs display a pattern of mixed activities between mono- and bicyclases, according to the molar ratio of γ-carotene to β-carotene [18,28,29], or δ-carotene to ε-carotene [30,31], whereas algal ε-cyclases that have been functionally tested at present are all monocyclases [32,33].

### 3.2. Divergence Times between LYCEs and LCYBs in Green Lineage

**Figure 3:**
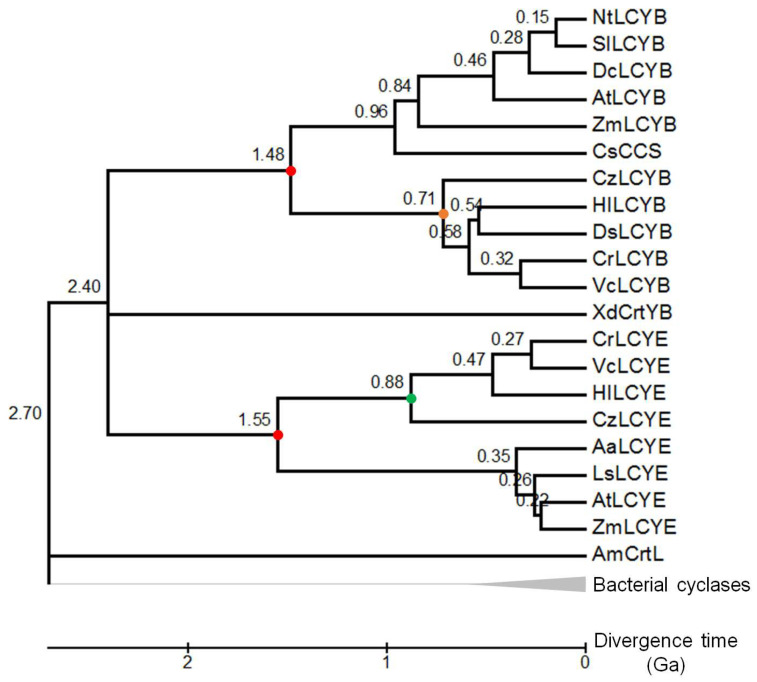
Divergence time of cyclases. The timetree is inferred based on the Bayesian tree by using the Reltime method and the General Time Reversible model. The cyanobacterial AmCrtY and algal CzLCYE are used as calibration with constraints ranging from 2.7 to 3.0 Ga. The estimated log likelihood value is −23,343.27. Nodes of green lineage LCYEs and LCYBs are highlighted by red-dotted cycles. Notes of algal LCYEs and LCYBs are highlighted by green- and orange-dotted cyclases, respectively.

The Bayesian tree inferred that LCYEs appeared earlier than LCYBs (Figure 2). To further infer the exact divergence time of each node, a time tree was constructed based on the emergence time of cyanobacteria (2.7 to 3.0 Ga) by using bacterial cyclases as the outgroup. As Figure 3 shows, green lineage LCYEs evolved at ~1.55 Ga, ~70 million years earlier than LCYBs, who evolved at ~1.48 Ga. Similarly, algal LCYEs also evolved earlier, at ~0.88 Ga, than algal LCYBs. Though we cannot compare plant LCYEs and LCYBs due to poor cyclase pairing availability, i.e., only Arabidopsis and maize cyclase pairs (AtLCYE and AtLCYB, and ZmLCYE and ZmLCYB) were used, making different ancestral nodes between plant LCYEs and LCYBs inferred, we believe similar results would be found in plant groups.

### 3.3. Role of Recombination and Selection

The role of selection was then determined using the Bayesian tree. To eliminate the interference of recombination to the analysis, a recombinant analysis was carried out. Only events with statistically significant difference detected by at least four of all the methods implemented in the software were considered recombinants. The results showed that one recombinant event probably occurred in maize LCYB (Figure 2 and Supplementary Table S4).

Then, selection analysis was performed. Site models did not find any site under positive selection; the free-ratio model revealed nine branches under very strong positive selection (ω much larger than 1), among which five branches showed statistical significance, e.g., branches B, F, G, H, and I, according to the likelihood ratio test (LRT) by two-ratio models (Supplementary Table S5). However, only branches B, G, H, and I showing significantly higher LRT than the one-ratio model had ω > 1 according to the two-ratio models (Supplementary Table S5). Branch B located at the clade of green lineage LCYEs, which was likely to evolve from cyanobacterial CrtL. Branch G contains *H. lacustris* HlLCYB and *Dunaliella salina* DsLCYB. Branch H comprises CrLCYE and VcLCYE. Branch I includes *Arabidopsis thaliana* LCYE (AtLCYE) and maize LCYE (ZmLCYE).

Further analysis using branch-site models for these four branches found many sites under strong positive selection (Supplementary Table S5). Up to 36 sites were found in the LCYE clade (branch B). Four sites under positive selection were found in branch G containing HlLCYB and DsLCYB. Branch H had 19 sites under positive selection. One site (T_86_ in HlLCYB) was found both in branch B and G, while two sites (A_210_ and A_468_ in HlLCYE) were found in branches B and H.

### 3.4. Functional Divergence between LCYEs and LCYBs

Since green lineage LCYBs seem to have evolved from LCYEs by gene duplication, analysis of type I and II functional divergences was carried out. Seven pairs of clusters were analyzed which include green lineage LCYEs/LCYBs, and six subgroups of algal LCYEs/plant LCYEs, algal LCYEs/algal LCYBs, algal LCYEs/plant LCYBs, plant LCYEs/algal LCYBs, plant LCYEs/plant LCYBs, and algal LCYBs/plant LCYBs. Only the green lineage group and the subgroup of plant LCYEs/algal LCYBs had *θ*_I_ values > 0 and showed statistically significant differences (Table 1). Furthermore, many sites were found, for instance, 16 sites in green lineage LCYEs/LCYBs and 46 sites in plant LCYEs/algal LCYBs, whereas no type II functional divergence was found because the *θ*_II_ values were negative or the analysis did not show a statistically significant difference.

### 3.5. Mapping Positive Selection Sites onto the Protein Structures

**Figure 4:**
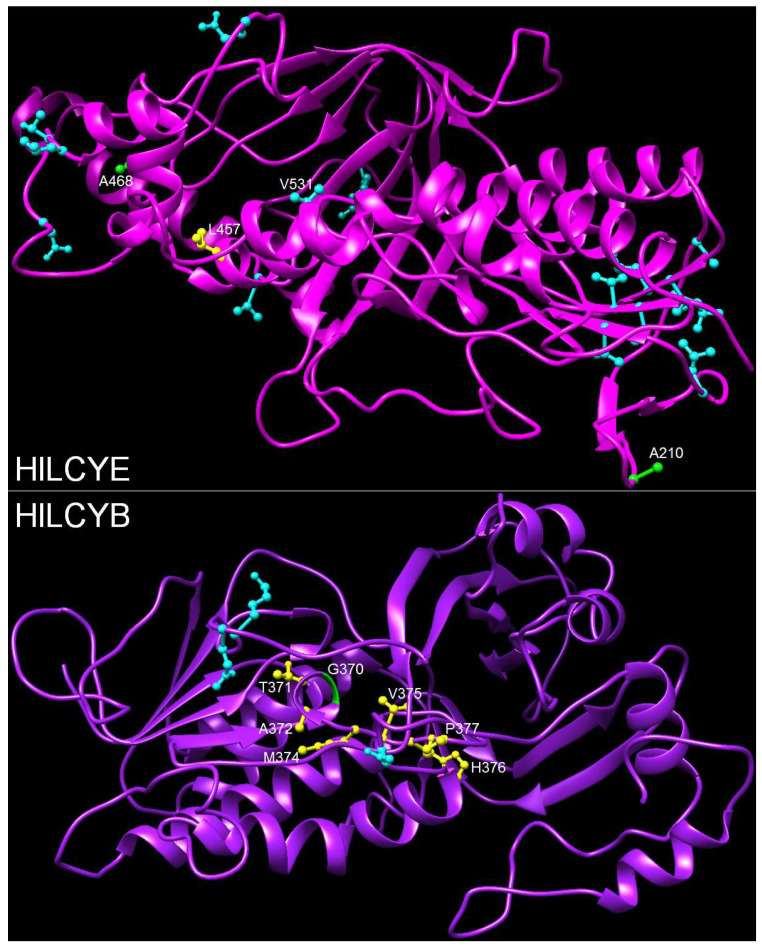
Sites under positive selection mapped onto the protein structures of *H. lacustris* cyclases. Sites detected in branch H (cyan ball and stick), sites (A_210_ and A_468_) detected in both branches B and H (green ball and stick), and L_457_ detected in branch B (yellow ball and stick), are mapped onto HlLCYE. Sites detected in branch G (cyan ball and stick), and G_370_ detected in branch B (green ribbon) as well as other sites of the GTAX_3_HP motif (yellow ball and stick), are mapped onto HlLCYB.

Sites under positive selection were mapped onto the 3D structures of HlLCYE and HlLCYB constructed by homology modeling (Figure 4). Site distribution in HlLYCE is largely on the antiparallel β-sheets and α-turns; only a few sites are on the α-helixes, which include A_468_ simultaneously detected in branch B and H, L_457_ found in branch B, and V_531_ found in branch H. These three sites are distinctive in that (1) they are located within the catalytic cavity; (2) L_457_ is at the homologous position (Supplementary Figure S1) to H_457_ of the bicyclase LsLCYE [30]. Three of four sites in branch G were mapped onto the structure of HlLCYB and they are all on the α-turn. G_370_ in branch B was mapped onto a short α-helix, which together with seven adjacent amino acids forms a distinct motif (GTAX_3_HP, X represents any amino acid, hereinafter inclusive) that is highly conserved among bicyclases but varies from the monocyclase CrtYm (TAGX_3_KA) (Supplementary Figure S1) in the catalytic cavity (Figure 4).

### 3.6. Positively Selected Sites Determine the Monocyclase Activity of HlLCYE

**Figure 5:**
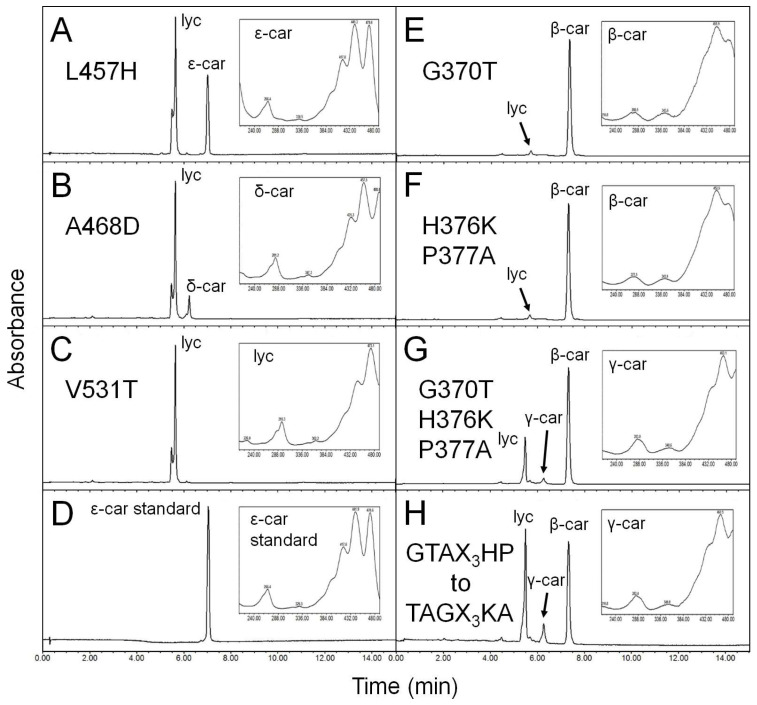
Conversions between the mono- and bicyclase activities of the *H. lacustris* cyclases by site-directed mutagenesis. (**A**–**C**) substitutions of L457H, A498S, and V531T in HlLCYE, respectively. (**D**) ε-carotenoid standard. (**E**–**H**) substitutions of G370T, H376K/P377A, G370T/H376K/P377A, and the whole motif (TAGX_3_KA) in HlLCYB, respectively. Absorption spectra of representative carotenoids shown in boxes are embedded into each panel, correspondingly.

The role of L_457_, A_468_, and V_531_ in controlling the monocyclase activity of HlLCYE, and the GTAX_3_HP motif in controlling the bicyclase activity of HlLCYB was investigated by site substitution with LsLCYE histidine, HlLCYB aspartate and threonine, and CrtYm TAGX_3_KA, respectively. An alternative profile of major pigments from δ-carotene to ε-carotene in the *E. coli* strain carrying the HlLCYE (L457H) mutant was observed (Figure 5A). Changing A_468_ to HlLCYB aspartate (A468D) remarkably reduced the amount of δ-carotene (Figure 5B), whereas replacing V_531_ with HlLCYB threonine (V531T) totally inactivated the catalytic activity of the enzyme (Figure 5C). L_457_ plays a key role in retaining the monocyclase activity of HlLCYE, while A_468_, and V_531_ are crucial for the catalytic activity. These results implied that positive selection events might have happened in an unknown ancestor, probably after the occurrence of cyanobacterial CrtL (sites A_468_ and in L_457_ branch B), to render a switch between mono- and bicyclase activities of HlLCYE (and/or other ε-monocyclases in green lineage), and to stabilize the catalytic cavity after its appearance (site V_531_ in branch H).

Single substitution of G_370_ by CrtYm threonine (Figure 5E) and double substitution of H376K and P377A by CrtYm lysine and alanine (Figure 5F) slightly lowered the catalytic activity of HlLCYB, while single substitution of other amino acids did not change the product profile (Supplementary Table S3). Simultaneous substitutions of G370T, H376K and P377A led to a small amount of γ-carotene and a considerable amount of lycopene, with β-carotene being the major product (Figure 5G). Substitution of the whole motif by that of CrtYm further enhanced γ-carotene accumulation; in this case, lycopene was the predominant product over β-carotene (Figure 5H). It seems that the positive selected G_370_ has little effect on the catalytic activity of HlLCYB.

## 4. Discussion

### 4.1. From Primary to Secondary Adaptations to Earth’s Oxygenation

Oxygenic photosynthesis in cyanobacteria led to great changes in the Earth’s system and the evolution of complex life (Figure 1). Evolution of the oxygenic photosystem in cyanobacteria was accompanied by the integration of antioxidant systems into photosynthetic apparatus, e.g., the primary carotenoid biosynthetic pathway, against harm by excess light radiation. The rapid increase in atmospheric oxygen concentrations forced a heterotrophic eukaryote to capture and integrate a cyanobacterium for survival, leading to the origin of the earliest oxygenic photosynthetic eukaryotes and subsequent diversification of green lineage at ~1.5 Ga [7], very close to our estimation that green lineage LCYEs probably evolved from cyanobacterial AmCrtL at ~1.55 Ga (Figure 3). As the oxygen content further rose to 18% PAL during the Mesoproterozoic, the primitive antioxidant systems obtained from cyanobacteria were likely strengthened in green lineage (Figure 2, branch B) through strong positive selection (ω = 999.0, 36 positively selected sites in Supplementary Table S5). The mutation of two positively selective sites (A_468_ and V_531_) in the catalytic cavity of HlLCYE (Figure 4) significantly lowered the activity and decreased the amount of product (Figure 5 and Supplementary Table S3). According to the origin of plasmid through the so-called primary endosymbiosis [7], we designate the development of these antioxidant systems as the primary adaptation to the first oxygenation of the Earth (the GOE), as evidenced by the phylogeny of green lineage LCYEs (Figure 2 and Figure 3).

As light-harvesting pigments, the major function of primary carotenoids is photosynthesis [10]. Our previous study also found that the in vivo amount of primary carotenoids was low under stress conditions, which was consistent with the lower activity of HlLCYE relative to HlLCYB, indicating the limited effect of photoprotection by primary carotenoids [19]. The low activity of LCYEs relative to LCYBs seems to be a general case in green lineage (Figure 2), as we found that the lycopene substrate was more or less left in many activity assays for LCYEs [30,31,32,33,34], while the substrate in those for LCYBs was used up [35,36,37,38]. More importantly, the antioxidant activity of primary carotenoids is intrinsically much lower than that of secondary carotenoids, especially astaxanthin [39]. Therefore, solely relying on the primary antioxidant systems is unsecured, especially as the oxygen levels steeply increased during the second oxygenation (the NOE) (Figure 1). These facts urge second systems with enhanced antioxidant activity.

However, the emergence of a secondary carotenoid biosynthetic system seems to have happened soon after the development of the primary carotenoid biosynthetic system, as the divergence time between LCYBs (1.48 Ga) and LCYEs (1.55 Ga) indicated (Figure 3). The close divergence time demonstrates that the origin of LYCBs is likely by gene duplication from LCYEs. Consequently, green lineage had to improve their ability to overcome sharply increasing oxygen based on extant antioxidant systems. For the outstanding antioxidant activity, the biosynthetic system of secondary carotenoids is naturally selected as an effective target. Strong positive selection, e.g., the branch (branch G, ω = 798.3) of HlLCYB and DsLCYB (Figure 2 and Supplementary Table S5), and type I functional divergence in green lineage LCYEs and LCYBs (Table 1) were detected. In addition, the second oxygenation also had selective pressure on the established primary carotenoid biosynthetic system since strong positive selection was detected in two branches (H and I) of LCYEs (Figure 2), though the effect was somewhat delayed (Figure 3). Collectively, we call these responses the secondary adaptation to the NOE. The generally enhanced activity of LCYBs/bicyclases versus the relatively low activity of LCYEs/monocyclases (as discussed below) indicates a historical trace from primary to secondary adaptations to the evolution of Earth’s oxygenation.

### 4.2. Adaptive Functionalization from Monocyclase to Bicyclase

**Figure 6:**
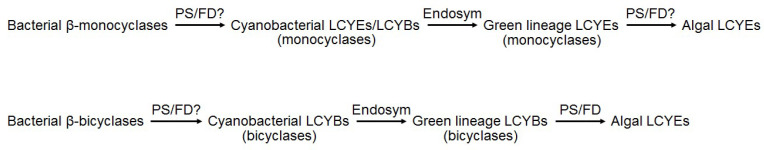
Hypothetic evolutionary mechanisms of contemporary algal LCYEs. PS: positive selection; FD: functional divergence; Endosym: endosymbiosis. Question mark indicates tentative processes.

At present, most LCYEs are monocyclase that introduce a single ε-ring to either end of lycopene to form δ-carotene, with exceptions in plants such as *Lactuca sativa* [40] and *Zea mays* [31] containing bicyclase LCYEs. However, to the best of our knowledge, a limited number of algal LCYEs that have already been functionally determined are all monocyclases [32,33]. From the view of evolution, green lineage LCYEs have a closer evolutionary distance to bacterial cyclases than LCYBs (Figure 2). It is widely accepted that green lineage originated from oxygenic photosynthetic cyanobacteria by primary endosymbiosis. And cyanobacteria probably evolved from anaerobic bacteria that conducted anoxygenic photosynthesis [41]. Therefore, it is possible that algal LCYEs evolved from an ancestor of cyanobacterial ε-/β-monocyclase stemming from bacterial monocyclase (Figure 6). The cyanobacterial AmCrtL used in this study is likely a β-monocyclase because its host contains chlorophyll d derived from monocyclic carotenoids as the major chlorophyll [42]. Recent studies also found cyanobacterial ε-/β-monocyclase in *Prochlorococcus* sp. MED4 [43] and *Synechocystis* sp. strain PCC 6803 [44]. Similar to green lineage LCYEs, these cyanobacterial monocyclases, together with most of bacterial monocyclases reported, exhibit relatively low catalytic activity [28,43,44,45,46,47]. Therefore, it could be inferred that the activity of the ancestor of cyanobacterial monocyclases may not be very high due to the relative low levels of oxygen at that time, leading to the universally low activity of green lineage LCYEs (Figure 2, Supplementary Table S3 and our previous study [19]). Because all anoxygenic photosynthetic bacteria known to date have no LCYE homologue [43], functional divergence and/or positive Darwinian selection should have occurred during the transformation from bacterial β-monocyclase to cyanobacterial ε-monocyclase. Further analyses are required to validate this hypothesis.

Another way from which green lineage LCYEs evolved follows the canonical cyanobacterial β-bicyclase that originated from bacterial β-bicyclases (Figure 6). In this way, functional divergence and/or positive selection should happen to transform enzyme activity from β-cyclase to ε-cyclase in green lineage after the primary endosymbiosis of cyanobacterial LCYBs. We did detect strong positive selection in branch B after green lineage LCYEs diverged from cyanobacterial LCYBs (Figure 2). Up to 36 sites were detected in this branch (Supplementary Table S5). In this scenario, however, algal LCYBs likely appeared earlier than LCYEs, i.e., algal LCYEs should have occurred by gene duplication from LCYBs rather than the opposite in our analysis (Figure 3). Seemingly, the origin of green lineage LCYEs is complex to unravel.

In contrast to LCYEs, most LCYBs are bicyclase with exceptions found in *Myxococcus xanthus* [46] and *Rhodococcus erythropolis* [29], which contain LCYBs with monocyclase activity that introduce only one β-ring to lycopene. Phylogenetic analysis showed an evolutionary trend from monocyclase to bicyclase in bacteria and algae (Figure 2) according to the molar ratio of γ-carotene to β-carotene [18,28,29]. It seems that evolution of bicyclases from monocyclases is a practical requirement by increasing oxygen levels because only from their product (β-carotene) can bicyclic secondary carotenoids possessing excellent antioxidant activity be synthesized [39]. During the transformation from LCYEs to LCYBs by gene duplication, functional divergence may be the main force (Table 1). After, positive selection may have an effect on some branches to accelerate the adaptation to increasing oxidative stress. For instance, the branch of HlLCYB and DsLCYB was likely under positive selection (Figure 2 and Supplementary Table S5). As a result, *H. lacustris* acquired a distinctive ability to accumulate astaxanthin. Replacing the positively selected G_370_ slightly lowered the activity of HlLCYB (Figure 5 and Supplementary Table S3), also implying such an effect.

The divergence between LCYEs and LCYBs, as well as differentiation of activity, i.e., weak monocyclase versus robust bicyclase, results in adaptive functionalization to survive in the current fully oxidized Earth: primary carotenoids as light-harvesting pigments participate in fundamental photosynthesis, while secondary carotenoids such as strong antioxidants are involved in photoprotection. Therefore, non-inductive primary lutein was maintained at a basal level at all conditions, and secondary astaxanthin was induced by stresses [19].

### 4.3. Oxidative Adaptation Has Been Widely Preserved

The current fully oxidized world requires extant organisms to develop strong resistance to oxidation. Several measures have been adopted, including aerobic respiration and a series of antioxidant enzymes such as superoxide dismutase and peroxidase [48,49]. For oxygenic photosynthetic organisms, it is wise to establish the first line of defense at the battlefront, the photosystems, against excess light radiation and oxidative stress. Carotenoids in chloroplast are selected as one kind of such defensive molecules, which marks the adaptively evolutionary trace to Earth’s oxygenation.

Stress-induced synthesis of secondary carotenoids is mediated by ROS [16]. Excess light irradiation leads to photoinhibition by the over-reduction of plastoquinone in photosystem II (PSII) and the formation of ROS in the reaction center of PSII [17]. Additionally, nutrient starvation and high salinity also lead to the formation of ROS [16]. Therefore, the accumulation of secondary carotenoids under environmental stresses, especially high light, is a protective strategy against ROS. As a representative, *H. lacustris* accumulated the maximum astaxanthin under oxidative stress, e.g., treatments of high light and FeSO_4_ + SA, ~54.3% of total astaxanthin [19]. High light-induced oxidative stress is among the strongest inducers for secondary carotenoids in a wide range of photosynthetic organisms, from green algae to higher plants [50,51]. Effective oxidative adaptation has been widely preserved in oxygenic photosynthetic organisms during Earth’s oxygenation.

## 5. Conclusions

This study has characterized two types of cyclases in the model *H. lacustris* elaborately adaptive to Earth’s oxygenation. The functional divergence in activity of the two cyclases is a consequence of primary and secondary adaptations to two consecutive events of oxygenation on Earth, indicating a common evolutionary process of oxygenic photosynthetic organisms. We propose a hypothetic evolutionary mechanism of monocyclase origination from anoxygenic photosynthetic bacterial β-monocyclases via cyanobacterial ε-monocyclases to green lineage ε-monocyclases. Further investigations on the evolution of cyanobacterial monocyclases are required to better verify this mechanism.

## Figures and Tables

**Table 1:** Type I functional divergence analysis.

	*θ*_I_ ± SE	LRT	*p* Value	Sites
green lineage LYCEs/LCYBs	0.44 ± 0.14	9.37	*p* < 0.002	HlLCYE: D130, M131, E154, D159, L172, H222, A238, R328, R353, D383, E410, T448, A459, L463, R482, S492 HlLCYB: G130, I131, I154, A159, F172, G222, R238, E328, P353, M383, Q410, G448, Q459, F463, R482, G492
algal LCYEs/plant LCYEs	0.38 ± 0.28	1.87	*p* > 0.05	
algal LCYEs/algal LCYBs	0.14 ± 0.31	0.2	*p* > 0.05	
algal LCYEs/plant LCYBs	0.33 ± 0.28	1.35	*p* > 0.05	
plant LCYEs/algal LCYBs	0.67 ± 0.33	4.17	*p* < 0.04	HlLCYB: P94, V96, R113, F119, S120, V121, C122, V124, L129, G130, E153, V155, P157, K158, A159, N165, L173, V180, R182, P183, S187, L201, A220, D221, G227, V237, A259, E260, E269, M271, M274, H288, P302, R307, A317, R318, A320, D331, A332, I339, E345, L351, P362, A389, V390, A432, P435, Q441, R442, L443, L451, L453, L457, Q459, D462, F463, F473, H476, F485, V512
plant LCYEs/plant LCYBs	0.25 ± 0.29	0.73	*p* > 0.05	
algal LCYBs/plant LCYBs	0.61 ± 0.37	2.69	*p* > 0.05	

Type I (*θ*_I_) functional divergence (±standard error (SE)) and LRT values for significance were estimated using DIVERGE3.0B1. Sites in HlLCYE are HlLCYB are shown.

## Data Availability

Data are contained within the article.

## Supplementary Materials

The following supporting information can be downloaded at: https://www.mdpi.com/article/10.3390/life14050576/s1, Figure S1: Multiple secondary domains in cyclases. Figure S2: Molecular evolutionary relationships of the *H. lacustris* cyclases by the Neighbor Joining method. Table S1: Primers used in this study. Table S2: Sequences used for sequence alignment and molecular evolution analyses. Table S3: Product profiles of the *H. lacustris* cyclases and site-directed mutants expressed in *E. coli*. Table S4: Recombination analysis of cyclases. Table S5: LRT of the models used in the PAML analysis.
