# Supplementary material for: Haematococcus lacustris Carotenogensis: A Historical Event of Primary to Secondary Adaptations to Earth’s Oxygenation
Source: Life (Basel). 2024 Apr 30;14(5):576. doi: 10.3390/life14050576 (PMC11121925; doi:10.3390/life14050576)
Supplement: Supplementary file 1 [file life-14-00576-s001.zip › life-2755331-supplementary.pdf]

Supplementary Figures

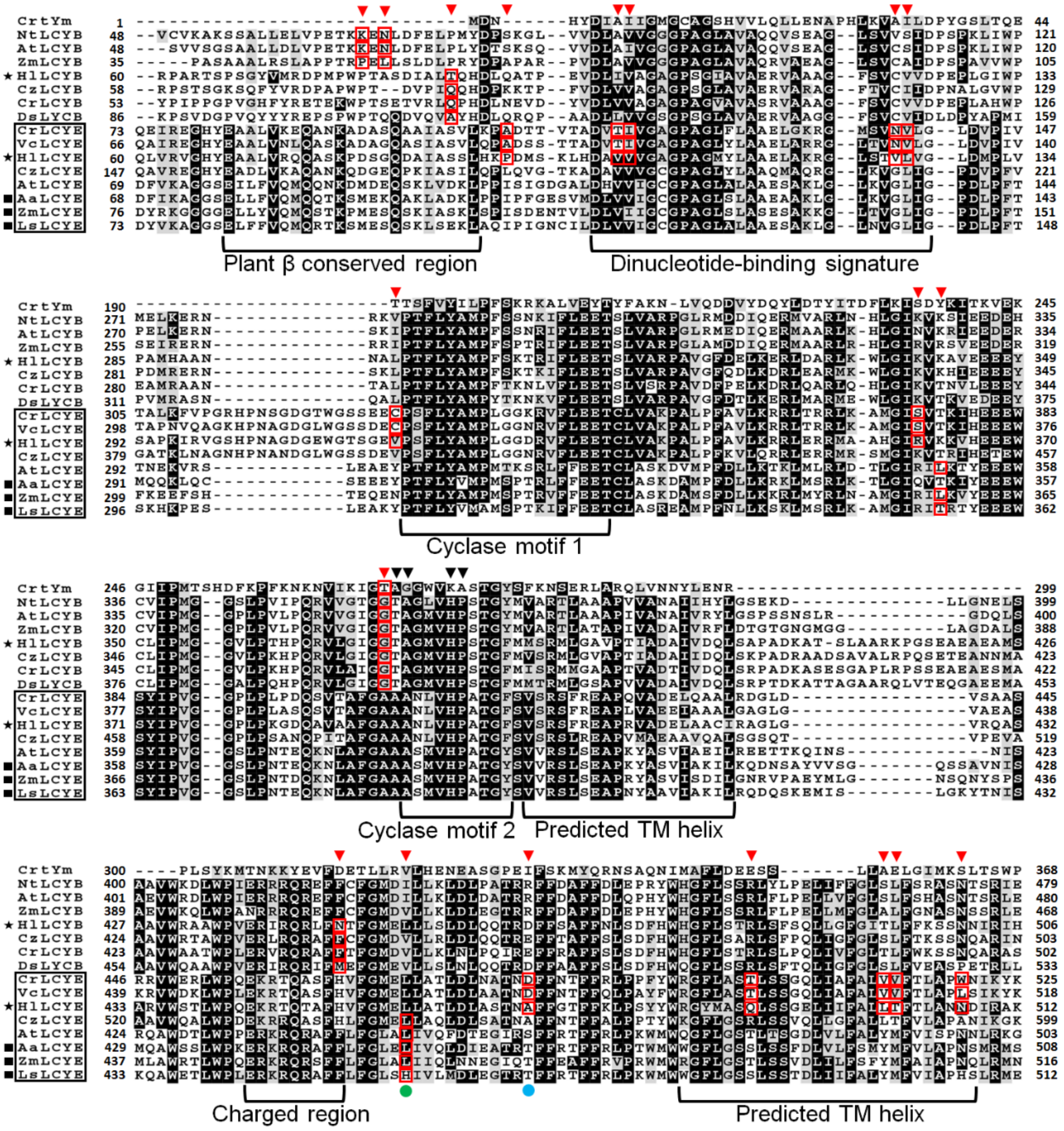

Figure S1. Multiple secondary domains in cyclases. The regulatory motif conserved in all β-bicyclases are highlighted by black arrowheads. Some positively selected sites are red-boxed and indicated by red arrowheads. The two residues varying between ε-mono- and bicyclases are highlighted by filled cycles. Filled rectangle indicated three ε-bicyclases. Stars indicate the HlLCYE and HlLCYB characterized in this study.

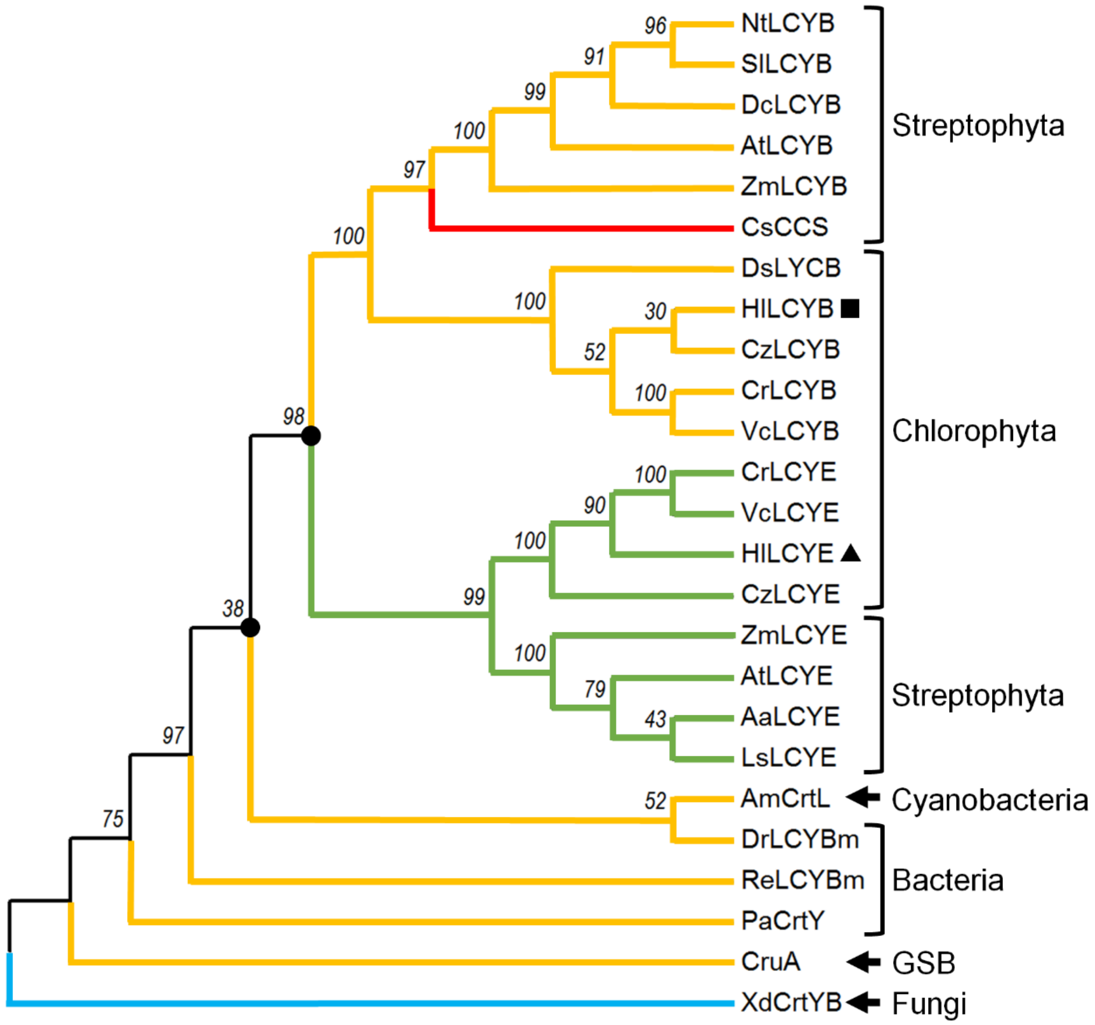

Figure S2. Molecular evolutionary relationships of the *H. lacustris* cyclases by the Neighbor-Joining method. The orange, green, red, and blue lines highlight LCYBs, LCYEs, CCS, and fungal bi-functional cyclase, respectively. The nodes of gene duplication during evolution are dotted. The bootstrap consensus tree inferred from 1000 replicates is taken to represent the evolutionary history of the taxa analyzed. Branches corresponding to partitions reproduced in less than 50% bootstrap replicates are collapsed. The percentage of replicate trees in which the associated taxa clustered together in the bootstrap test (1000 replicates) are shown above the branches.

Supplementary Tables

Table S1. Primers used in this study.

Manipulation	Primer	Sequence (5' to 3')
**Expression plasmid construction**		
pACCRT-EIB-B	EIBYb F	TACCGCATTA***AAGCTT***aggaggattacaaa**ATG**TTGAGCAAACAACACGATT
	EIBYb R	TATCATCGATA***AGCTT***CTACTTGATCATGGCTGGCG
pACCRT-EIB-E	EIBYe F	TACCGCATTA***AAGCTT***aggaggattacaaa**ATG**CAACCACTGCGTGTAGCAAC
	EIBYe R	TATCATCGAT***AAGCTT***TTAGGCCTGGTTCGGCTCCA
**Site-directed mutagenesis**		
	SP[b]	CCACTGGCAG***ACTAGT***GTAACAGGATTAGC
LCYE mutants	L457H	ATCCAGAGTGGCCAGATGCTCCATGCCAAATAC
	A498S	AGTGAAAGTCAGCAGGCTGAAGATGATCAGCTC
	L457H/A498S[c]	AGTGAAAGTCAGCAGGCTGAAGATGATCAGCTC
	TAGX3KA	CATGAACCCTGTTGAGGCCTTCACCATGCCGCCCGCCGTGCCAATGCCCAGCAC
	G370T	CACCATGCCCGCCGTCGTGCCAATGCCCAGCAC
	T371A	ATGCACCATGCCCGCCGCGCCGCCAATGCCCAG
LCYB mutants	A372G	GGGATGCACCATGCCGCCCGTGCCGCCAATGCC
	T371A/A372G[c]	GGGATGCACCATGCCGCCCGTGCCGCCAATGCC
	H376K	GAACCCTGTTGAGGGCTTCACCATGCCCGCCGT
	P377A	CATGAACCCTGTTGAGGCATGCACCATGCCCGC
	H376K/P377A[3]	CATGAACCCTGTTGAGGCATGCACCATGCCCGC

Sequences overlapping those of vectors are underlined; restriction sites are shown in bold italics; initiation codon (ATG) artificially added is shown in bold; mutant sites are shown in red. The selection primer (SP) was designed according to the user protocol.

Table S2. Sequences used for sequence alignment and molecular evolution analyses.

**Type**	**Species**	**Accession number**
Streptophyta LCYB	*Nicotiana tabacum*	NP_001311716
	Arabidopsis thaliana	AAB53337
	Zea mays	AAO18661
	Solanum lycopersicum	ABR57232
	Daucus carota	NP_001316089
Chlorophyta LCYB	*Haematococcus lacustris*	KX424526 (this study)
	Chlamydomonas reinhardtii	AAX54906
	Chromochloris zofingiensis	CBH31263
	Dunaliella salina	ACA34344
	Volvox carteri f. nagariensis	EFJ41647
Streptophyta LCYE	*Arabidopsis thaliana*	AAB53336
	Adonis aestivalis	AAK07431
	Zea mays	NP_001146840
	Lactuca sativa	AAK07434
Chlorophyta LCYE	*Chlamydomonas reinhardtii*	XP_001696529
	Volvox carteri f. nagariensis	XP_002945935
	Haematococcus lacustris	AKT95178 (this study)
	Chromochloris zofingiensis	CCG06343
Bacterial β-monocyclase	Unclassified *Flavobacterium*	BAC77673
	Rhodococcus erythropolis	AAR98749
	Deinococcus radiodurans	AAF10377
Cyanobacterial CrtL	*Acaryochloris marina*	WP_012166204
Eubacterial CrtY	*Pantoea ananatis*	ADD79327
Fungal CrtB	*Xanthophyllomyces dendrorhous*	AAY33923
Streptophyta CCS	*Citrus sinensis*	AAF18389
GSB CruA	*Chlorobium tepidum*	NP_661357.1

CCS: capsanthin-capsorubin synthase

Table S3. Product profiles of the *H. lacustris* cyclases and site-directed mutants expressed in *E. coli*.

	Percentage of total carotenoids (n = 3)								
	lyc	δ-car	γ-car	ε-car	α-car	β-car	neur	α-zeacar	β-zeacar
EIB-E	67.89 ± 2	32.11 ± 3	n.d.	n.d.	n.d.	n.d.	n.d.	n.d.	n.d.
EIB-B	1.08 ± 1	n.d.	n.d.	n.d.	n.d.	98.92 ± 4	n.d.	n.d.	n.d.
**Mutants of HlLCYE**									
L457H	66.52 ± 0.5	n.d.	n.d.	33.48 ± 2	n.d.	n.d.	n.d.	n.d.	n.d.
A468D	87.7 ± 3	12.3 ± 1	n.d.	n.d.	n.d.	n.d.	n.d.	n.d.	n.d.
V531T	99.83 ± 2	0.17 ± 0.1	n.d.	n.d.	n.d.	n.d.	n.d.	n.d.	n.d.
**Mutants of HlLCYB**									
G370T	2.22 ± 0.5	n.d.	n.d.	n.d.	n.d.	97.78 ± 4	n.d.	n.d.	n.d.
T371A	1.24 ± 0.5	n.d.	n.d.	n.d.	n.d.	98.76 ± 2	n.d.	n.d.	n.d.
A372G	1.27 ± 0.2	n.d.	n.d.	n.d.	n.d.	98.73 ± 2	n.d.	n.d.	n.d.
H376K	1.21 ± 0.3	n.d.	n.d.	n.d.	n.d.	98.79 ± 1	n.d.	n.d.	n.d.
P377A	1.19 ± 0.5	n.d.	n.d.	n.d.	n.d.	98.81 ± 3	n.d.	n.d.	n.d.
T371A/A372G	1.39 ± 1	n.d.	n.d.	n.d.	n.d.	98.61 ± 1	n.d.	n.d.	n.d.
H376K/P377A	2.87 ± 1	n.d.	n.d.	n.d.	n.d.	97.13 ± 1	n.d.	n.d.	n.d.
G370T/H376K/P377A	21.91 ± 1	n.d.	3.57 ± 1	n.d.	n.d.	74.52 ± 2	n.d.	n.d.	n.d.
GTAX_3HP/TAGX_3KA	53.57 ± 3	n.d.	14.94 ± 2	n.d.	n.d.	31.49 ± 2	n.d.	n.d.	n.d.

Data are expressed as dry cell weight (DCW) percentages of total carotenoids in the *E. coli* strains, and each value is the mean ± SD for three experiments (n = 3). Lyc: lycopene; δ-car: δ-carotene; γ-car: γ-carotene; ε-car: ε-carotene; α-car: α-carotene; β-car: β-carotene; neur: neurosporene; α-zeacar: α-zeacarotene; β-zeacar: β-zeacarotene; n.d.: not detected.

Table S4. Recombination analysis of cyclases

	Breakpoint		Parent		Detection Methods								
Recombinant	Begin	End	Minor	Major	RDP	GENECONV	Bootscan	Maxchi	Chimaera	SiSscan	PhylPro	LARD	3Seq
ZmLCYB	1031	1365	AtLCYB	CrLCYB/VcLCYB	NS	0.0007797	NS	2.06E-02	1.65E-02	1.44E-09	NS	2.91E-02	NS

NS: No significant *P* value is recorded for this recombination event using this method.

Table S5. LRT of the models used in the PAML analysis

Model	*np*	Estimates of parameters	ln*L*	LRT pair	*df*	2Δln*L*	Positive selected sites
**Site models**							
M0	49	ω=0.07152	-33663.366682				
M3	53	p_0=0.38470, p_1=0.44096, (p_2=0.17434), ω_0=0.01935, ω_1=0.09159, ω_2=0.29940	-32903.382604	M0/M3	4	1519.9700**	
M1a	50	p_0=0.86750, (p_1=0.13250), ω_0=0.07646, (ω_1=1.00000)	-33424.440636				
M2a	52	p_0=0.86750, p_1=0.01616, (p_2=0.11635), ω_0=0.07646, (ω_1=1.00000), ω_2=1.00000	-33424.440636	M1a/M2a	2	0	
M7	50	p=1.12383, q=9.31286	-32906.043654				
M8	52	p_0=0.99027, p=1.15604, q=9.95730, (p_1=0.00973), ω=1.00000	-32904.834280	M7/M8	2	2.4188	
**Branch models**							
Fr	95	See **Figure 4**	-33550.427866	M0/Fr	46	225.8780**	
Ta	50	ω_0=0.07145, **ω_a=447.95515**	-33663.294828	M0/Ta	1	0.1437	
Tb	50	ω_0=0.07145, **ω_b=3.41633**	-33660.058438	M0/Tb	1	6.6165**	
Tc	50	ω_0=0.07152, **ω_c=287.71757**	-33663.291103	M0/Tc	1	0.1512	
Td	50	ω_0=0.07161, **ω_d=999.00000**	-33662.259393	M0/Td	1	2.2146	
Te	50	ω_0=0.07206, ω_e=0.00184	-33662.761843	M0/Te	1	1.2097	
Tf	50	ω_0=0.07700, ω_f=0.00587	-33653.210680	M0/Tf	1	20.3120**	
Tg	50	ω_0=0.07216, **ω_g=999.00000**	-33660.221006	M0/Tg	1	6.2914**	
Th	50	ω_0=0.07112, **ω_h=999.00000**	-33660.722873	M0/Th	1	5.2876*	
Ti	50	ω_0=0.06958, **ω_i=999.00000**	-33657.758291	M0/Ti	1	11.2168**	
**Branch-site models**							
A1b	51	p_0=0.68674, p_1=0.10482, (p_{2a}=0.18083, p_{2b}=0.02760), ω_0=0.07585, (ω_1=1.00000), b: ω_{2a}=0.07585, ω_{2b}=1.00000, f: ω_{2a}=1.00000, ω_{2b}=1.00000	-33418.029102				

Ab	52	p_0=0.66662, p_1=0.10212, (p_{2a}=0.20054, p_{2b}=0.03072), ω_0=0.07672, (ω_1=1.00000), b: ω_{2a}=0.07672, ω_{2b}=1.00000, f: **ω_{2a}=921.69928**, **ω_{2b}=921.69928**	-33412.910439	A1b/Ab	1	10.2373**	HlLCYE<br>S89*, Y112*, K119*, G128*, D130*, P132*, V134**, K145*, H151*, S180*, V209**, **A210***, T216*, A228*, T232**, L233*, A235*, A237*, P250*, S254*, E261*, F271**, T286*, K339*, H366*, Y372*, V375*, G382*, D383**, A391*, S403*, ***L457****, A459*, S465*, **A468***, A498*	HlLCYB<br>**T86***, A109*, A116*, D125*, E127*, I131*, P133**, E144*, E191*, D181*, V208**, N209*, S215*, G227*, L231**, D232*, T234*, H236*, F247*, Y251*, V258*, F266**, T281*, R318*, E345*, L351*, M354*, H361*, P362**, ***G370****, M382*, L451*, L453*, Q459*, D462*,G492*	
A1f	51	p_0=0.82918, p_1=0.12680, (p_{2a}=0.03818, p_{2b}=0.00584), ω_0=0.07576, (ω_1=1.00000), b: ω_{2a}=0.07576, ω_{2b}=1.00000, f: ω_{2a}=1.00000, ω_{2b}=1.00000	-33422.509336						
Af	52	p_0=0.83668, p_1=0.12892, (p_{2a}=0.02981, p_{2b}=0.00459), ω_0=0.07596, (ω_1=1.00000), b: ω_{2a}=0.07596, ω_{2b}=1.00000, f: **ω_{2a}=21.31733**, **ω_{2b}=21.31733**	-33417.738928	A1f/Af	1	9.5408*	AtLCYB<br>K65**, N67*, N494*		
A1g	51	p_0=0.53134, p_1=0.08186, (p_{2a}=0.33516, p_{2b}=0.05163), ω_0=0.07470, (ω_1=1.00000), b: ω_{2a}=0.07470, ω_{2b}=1.00000, f: ω_{2a}=1.00000, ω_{2b}=1.00000	-33416.077226						
Ag	52	p_0=0.81800, p_1=0.12905, (p_{2a}=0.04574, p_{2b}=0.00722), ω_0=0.07527, (ω_1=1.00000), b: ω_{2a}=0.07527, ω_{2b}=1.00000, f: **ω_{2a}=999.00000**, **ω_{2b}=999.00000**	-33406.684493	A1g/Ag	1	18.7855**	HlLCYB<br>**T86****, K242*, Q245*, N445*	DsLCYB<br>A112**, K268*, Q271*, M472*	
A1h	51	p_0=0.74005, p_1=0.11651, (p_{2a}=0.12393, p_{2b}=0.01951), ω_0=0.07480, (ω_1=1.00000), b: ω_{2a}=0.07480, ω_{2b}=1.00000, f: ω_{2a}=1.00000, ω_{2b}=1.00000	-33410.003873						
Ah	52	p_0=0.79428, p_1=0.13049, (p_{2a}=0.06461, p_{2b}=0.01062), ω_0=0.07616, (ω_1=1.00000), b: ω_{2a}=0.07616, ω_{2b}=1.00000, f: **ω_{2a}=999.00000**, **ω_{2b}=999.00000**	-33388.535879	A1h/Ah	1	42.9360*	HlLCYE<br>P93*, V102**, V103*, V125**, L126**, A198*, L200**, **A210***, L226**, V231**, S251*, V315**, R361**, **A468****, Q488*, L500*, T501**, N507**, V531**	CrLCYE<br>A106*, T115**, I116*, N138**, V139**, R211*, S213**, V223*, Y239**, T244**, I264*, C328**, S374**, D481**, T501*, V513*, V514**, W520**, Q544**	VcLCYE<br>A99*, T108**, I109*, N131**, V132**, R204*, S206**, V216*, Y232**, T237**, I257*, C321**, S467**, D464**, T494*, V506*, V507**, L513**, Q537**
A1i	51	p_0=0.57455, p_1=0.08544, (p_{2a}=0.29600, p_{2b}=0.04401), ω_0=0.07413, (ω_1=1.00000), b: ω_{2a}=0.07413, ω_{2b}=1.00000, f: ω_{2a}=1.00000, ω_{2b}=1.00000	-33419.336472						
Ai	52	p_0=0.80198, p_1=0.11853, (p_{2a}=0.06926, p_{2b}=0.01024), ω_0=0.07368, (ω_1=1.00000), b: ω_{2a}=0.07368, ω_{2b}=1.00000, f: **ω_{2a}=999.00000**, **ω_{2b}=999.00000**	-33417.040223	A1i/Ai	1	4.5925*	AtLCYE<br>L351*	ZmLCYE<br>L358*	LsLCYE<br>T355*

Selection analysis by three kinds of models is performed using Codeml implemented in PAML. *np*: number of free parameters. ln*L*: log

likelihood. LRT: likelihood ratio test. *df*: degrees of freedom. 2ΔIn*L*: twice the log-likelihood difference of the models compared. The significant tests at 5% cutoff are labeled with * and at 1% cutoff are labeled with **. Sites of HILCYE detected in both branches B and H are showed in bold. Sites of HILCYB detected in both branches B and G are also showed in bold. G_{370} of HILCYB and L_{457} of HILCYE detected in branch B is highlighted by italic bold.
